# Study of an Additional Layer of Cement Mantle Hip Joints for Reducing Cracks

**DOI:** 10.3390/jfb10030040

**Published:** 2019-09-06

**Authors:** J. Jamari, Eko Saputra, Iwan Budiwan Anwar, Emile van der Heide

**Affiliations:** 1 Department of Mechanical Engineering, Engineering Faculty, Diponegoro University, Jl. Prof. Soedarto SH Tembalang, Semarang 50275, Indonesia; 2 Laboratory for Surface Technology and Tribology, Engineering Technology Faculty, Twente University, Postbox 217, 7500 AE Enschede, The Netherlands (I.B.A.) (E.H.); 3 Orthopaedic and Traumatology Department, Prof. dr. R. Soeharso Orthopaedic Hospital, Jl. A. Yani Pabelan, Surakarta 57162, Indonesia

**Keywords:** additional layer, liner, cement mantle, hip joint

## Abstract

Failure of the cement mantle in total hip arthroplasty is not a simple phenomenon. Cracking, which can be caused by crack initiation and repeated loading, can cause loosening of the acetabular liner component. A previous study showed that addition of a metal layer between the liner and acetabular could reduce the stress at the contact surface of the cement mantle. This study elaborates on the performance of the additional layer. Several material properties of the layer were simulated using finite element analysis for maximum performance. A static contact analysis was used to simulate the stresses at the contact surface of the cement mantle. The results show that an additional layer of cobalt chrome produced the best performance.

## 1. Introduction

**Figure 1:**
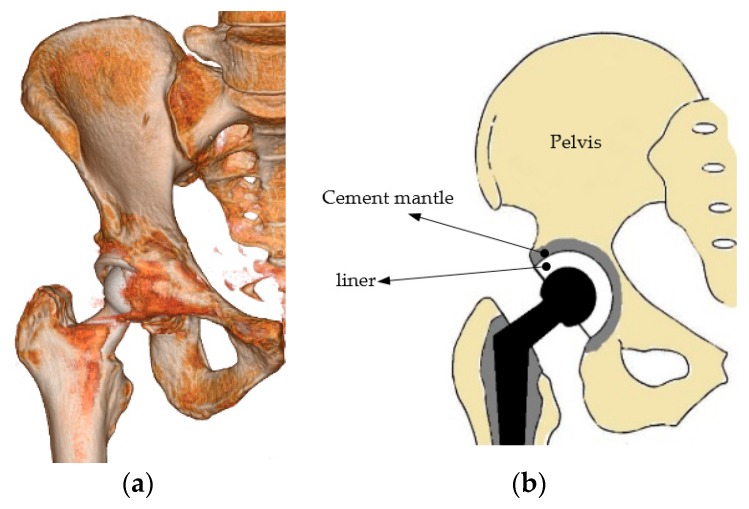
Cemented hip prosthesis: (**a**) three-dimensional illustration [5] and (**b**) illustration of the cross–section.

A cement mantle or bone cement is widely used as an implant material in cemented hip protheses [1]. Repeated cyclic loads may contribute to the failure of the mantle in the hip prosthesis [2]. Figure 1a,b shows the three-dimensional illustration of a cemented model of a hip prosthesis and a cross–sectional view. The cement is positioned between the pelvis and acetabular, and the performance of the cement mantle is indicated by cracking. A crack in the cement mantle indicates that the cement mantle has a defect or failure has occurred [3]. A less optimal thickness will produce higher stress on the cement mantle. Material defects due to initial cracking and high stress of contact loading will trigger the initial crack, and finally affect the crack in the cement mantle. Experimental fatigue tests or computer simulations can investigate the cracking problem [3,4].

A number of attempts have been performed by researchers to minimize the contact pressure or stresses on the cement mantle. Increasing the thickness of the cement mantle is of interest, and the effect of the thickness of PMMA (Poly(methyl methacrylate)) cement mantle on the strains of stem components in total hip replacement was investigated experimentally by Fisher et al. [1]. The variation of the thickness was conducted on two stem components. Axial loads that refer to walking and standing conditions were given to the stem components. Strains were measured by strain gauges embedded inside the cement mantle. The results showed that by increasing the thickness of the cement mantle by 1.3 mm thickness, the cement mantle strain could be reduced by approximately 40–49% [1]. 

Two different hip prostheses were studied by Ramos and Simoes for examining fatigue damage on a cement mantle as a function of thickness [6]. They concluded that the thickness of the cement mantle is a dominant factor for hip substitution. Numerical analysis was performed by Lamvohee et al. [7] to explore variables related to the thickness of a cement mantle such as the size of acetabular, body mass index, and quality of the bone. By increasing the thickness of the cement mantle, the maximum tensile stress decreases. Based on the aforementioned studies, better performance will be attained by increasing the cement mantle thickness. However, the study of Mann et al. [8,9] showed that the thickness of a cement mantle has no relation to the rate of fatigue crack growth.

For minimizing stress and contact pressure on the cement mantle, Jamari et al. [10] proposed adding a layer between the cement mantle and liner. Static contact simulations were performed to study the influence of the additional layer on the contact pressure and stress. The effect of repeated or cyclic contact loading has also been studied [11,12,13]. The effect of a repeated load may lead to hip prosthesis failure. The presence of an additional layer could reduce the stress on the cement mantle as reported in [10], which in turn, prevents the failure of the hip prosthesis system. By adding a Stainless SS316L layer between the liner and cement, the stresses and displacement decreased by more than 50%. The next question is what material will produce the best performance. This study will further explore the effect of the additional layer material as a function of stresses and displacement. Several materials will be simulated using finite element analysis.

## 2. Method

### 2.1. Model of Geometry and Material

**Figure 2:**
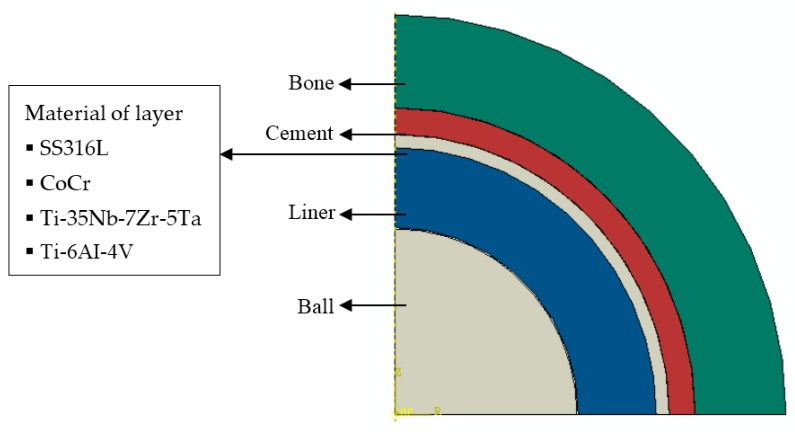
Geometric model of a hip prosthesis with the added layer.

Static contact analysis of the hip prosthesis system was conducted to generate stresses and deflection on the surface of the cement mantle. The finite element analysis software ABAQUS 6.12, was selected to analyze this case [14]. The cement mantle hip joint model consisted of bone, cement, liner, and head (ball) as depicted in Figure 1a. The ball is connected to the stem. The arrangement of the ball, liner, cement mantle, and bone is detailed in Figure 1b in the cross–sectional view. Due to the spherical geometry, an axisymmetrical model was employed for simulating the contact of the hip prosthesis system, see Figure 2. The geometric model of the contact system of a hip prosthesis with the added layer consists of bone, cement, the added layer, liner, and ball. The bone has a diameter of 60.2 mm and the ball has a diameter of 28 mm. The thickness of cement mantle used by Gun et al. [15] was adopted for calculating contact parameters in this study.

Table 1 summarizes the material properties from literature that were used for the components of the hip prosthesis simulation. According to Anderson et al. [16], Sahli et al. [17], and Ouinas et al. [18], cortical bone has a modulus of elasticity of 17,000 MPa and Poisson’s ratio of 0.3. The cement mantle of polymethyl methacrylate (PMMA) has a modulus of elasticity ranging from 2000 to 2300 MPa and Poisson’s ratio of 0.3 according to [17,18], and Achour et al. [19]. The ultra–high–molecular–weight polyethylene (UHMWPE) liner as used by [18,19], and Eichmiller et al. [20], has an elasticity modulus of 690 to 945 MPa and Poisson’s ratio of 0.45. The ball was 316 L stainless steel as used by Yildiz et al. [21], with a modulus of elasticity of 193,000 MPa and Poisson’s ratio of 0.3. In this simulation, the material properties of the addition layer varied cobalt chrome (CoCr), low module titanium beta alloy (Ti-35Nb-7Zr-5Ta), and titanium alloy (Ti-6AI-4V), as shown in Table 1. The moduli of elasticity of CoCr, Ti-35Nb-7Zr-5Ta, and Ti-6AI-4V are 230,000 MPa, 113,800 MPa, and 110,000 MPa, respectively [22,23].

### 2.2. Simulation Procedure

**Figure 3:**
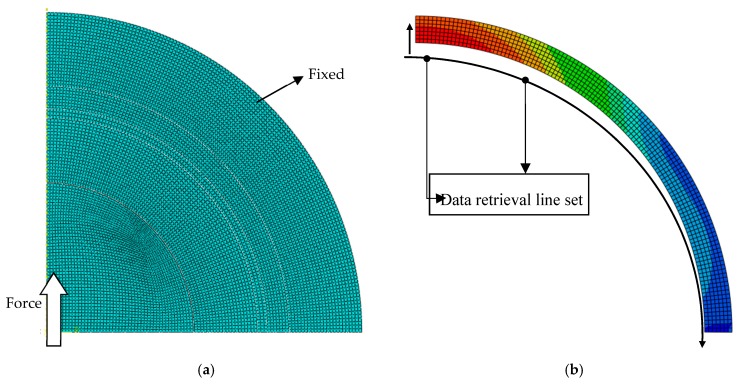
(**a**) The conditions of load, mesh, and boundary. (**b**) Data retrieval line set for the liner and PMMA.

Figure 3a shows the applied load and mesh in the hip prosthesis model. A static force of 3000 N was applied to the center of the ball following the model of Bergmann et al. [24] for hip contact forces under routine activities. The symmetrical boundary conditions were applied so that a quarter of the ball was produced. The model of the bone was attached to the outer surface. Contact occurs only between the liner’s inner surface and the ball surface. A bilinear axisymmetric structure of four quadrilateral nodes, reduced integration, and an hourglass control (CAX4R) mesh were employed for simulating the proposed contact model. The number of elements and nodes were about 8000.

The contact stress or contact pressure, von Mises stress, and deflection were observed. The contact stress was calculated to explore the distribution of stress on the area of the cement mantle surface. The von Mises failure criterion was applied for investigating the stresses along the thickness of the cement mantle. Deflection analysis was also performed to study the effect of a new layer on the stiffness of the cement mantle system. The location of the data collection is shown in Figure 3b. Data was taken from the retrieval line set for each node. 

## 3. Results and Discussion

**Figure 4:**
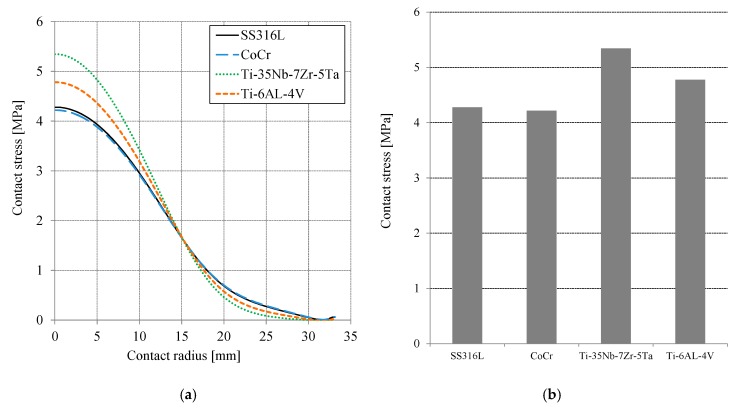
(**a**) Contact stress distribution on the cement mantle surface as a function of the contact radius and (**b**) maximum contact stress for all material variations.

Figure 4a presents the contact stress or contact pressure distribution on the surface of the cement mantle as a function of the contact radius. The S22 terminology of the ABAQUS function for post-processing was employed to demonstrate the contact stress on the cement mantle surface. For all simulated materials, the maximum contact stress location was at the center of the cement mantle. The contact stress decreases with the increase of the contact radius following the general contact stress distribution. Figure 4b shows the maximum contact stress for all simulated materials. The highest contact stress of the cement mantle occurred when Ti-35Nb-7Zr-5Ta was used, i.e., approximately 5.34 MPa. When using CoCr, the lowest contact stress occurred, i.e., approximately 4.2 MPa, for a difference of approximately 21%. Compared to SS316L, Ti-6AL-4V, and Ti-35Nb-7Zr-5Ta materials, the CoCr material is the best for reducing the contact stress, especially at the center of the contact area. The modulus of elasticity of the added layer plays an important role in the resulting contact stress. The lowest contact stress was attained when the highest elasticity modulus of the layer was employed. 

**Figure 5:**
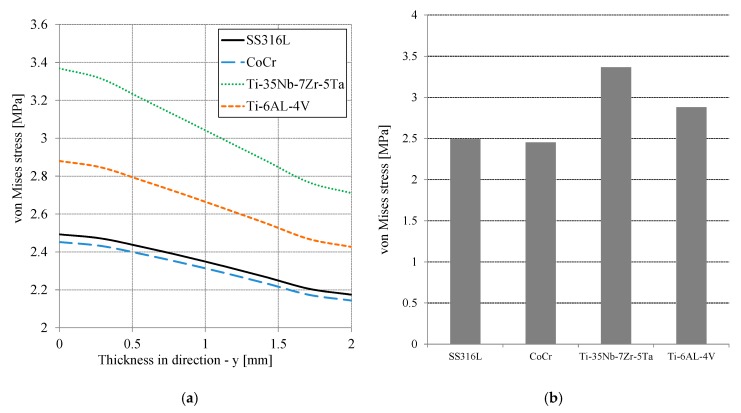
(**a**) Distribution of the von Mises stress of the cement mantle as a function of the thickness in the direction [−y] for all material variations and (**b**) the maximum von Mises stress as a function of the material.

Figure 5a presents the von Mises stress distribution on the cement mantle as a function of thickness or cement mantle depth. The highest von Mises stress occurred at the cement mantle contact and decreased with increasing thickness. The difference between the maximum and minimum of the von Mises stress along the thickness was about 14%. For all simulated materials, Ti-35Nb-7Zr-5Ta provided the highest von Mises stress, approximately 3.36 MPa, while CoCr produced the lowest von Mises stress of about 2.45 MPa. The Ti-6AL-4V material was slightly less than Ti-35Nb-7Zr-5Ta and the SS316L was slightly greater than CoCr. Figure 5b shows the highest stress of von Mises on the surface of the cement mantle for all simulated materials. The results showed that the CoCr material was best for reducing the maximum von Mises stress compared to SS316L, Ti-6AL-4V, and Ti-35Nb-7Zr-5Ta. However, the maximum von Mises stress for all simulated materials were still within the elastic limit over all thickness variations. The tensile strength of the PMMA material is approximately 25 MPa [17,18,19]. The von Mises stress results in this study confirms the study of stress [7] and strain [1] characteristics.

**Figure 6:**
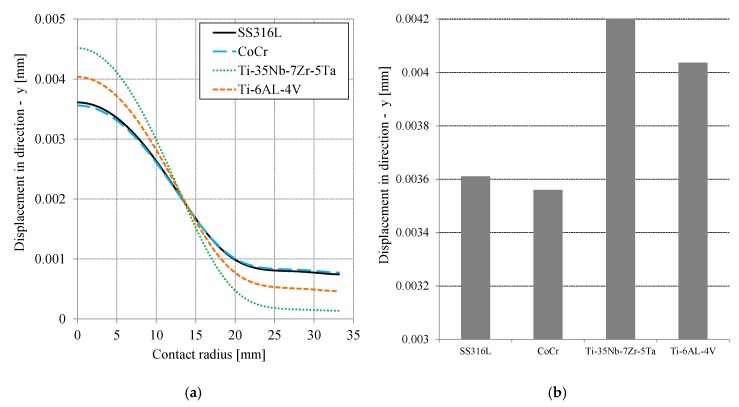
(**a**) Distribution of displacement on the cement mantle as a function of contact radius for all material variation and (**b**) the maximum displacement as a function of material.

Contact stress or contact pressure and von Mises stress are important for analyzing the contact system parameters. The deformation or displacement of the cement mantle, relative to the ball, is also crucial in analyzing the additional layer of the hip joint prosthesis system. Figure 6a describes the movement of the cement mantle in the −y direction, depending on the contact radius. U2 displacement in the ABAQUS post-processing feature was used to show the cement mantle displacement. The peak displacement was at the center of the PMMA (cement mantle). The curves of displacement were similar, following the curve of contact stress (pressure). The highest deformation of the cement mantle occurred with Ti-35Nb-7Zr-5Ta, i.e., about 0.00452 mm. The lowest deformation of the cement mantle occurred with CoCr, i.e., about 0.0037 mm. Figure 6b shows the maximum displacement of the cement mantle for all simulated materials. The results show that CoCr was best for reducing the displacement. The difference between the displacement of Ti-35Nb-7Zr-5Ta and CoCr was about 21%. For all the materials used, the characteristic that was most different was the magnitude of the elasticity modulus. The modulus of elasticity is related to the hardness of the material: the higher the modulus of elasticity, the higher the hardness. Therefore, CoCr provided the best performance (see Table 1).

Distribution of the contact pressure, von Mises stress, and displacement results for all simulated materials are similar to previous studies [10,11,12,13]. There is no strange distribution and the difference is only in the magnitude. The results are promising and the approach outlined in this study should be further explored for the effect of surface interactions between the layers [25,26] or the surface roughness [27].

Potential toxicity could also be interesting to study. Keegan et al. [28] reported on orthopedic metals and their potential toxicity in the arthroplasty patient. Several potentially harmful effects may occur in the blood, the immune system, the liver, the kidney, the respiratory system, the nervous system, the heart and vascular systems, the musculoskeletal system, the endocrine system, the visual and auditory systems, the skin, etc. However, in this study, the additional layer is located between the liner and the bone. Therefore, there is no friction and no wear debris, which might cause potential toxicity. 

## 4. Conclusions

Static simulation of contact on the hip joint prosthesis system with material variations of an additional layer was conducted using finite element analysis software. SS316L, CoCr (cobalt chrome), Ti-35Nb-7Zr-5Ta, and Ti-6AI-4V varied the material properties of the additional layer, and the thickness of the cement mantle was constant. The contact stress (pressure), von Mises stress, and deflection of the cement mantle were studied to analyze the effect of the material variation. The highest contact stress of the cement mantle occurred when Ti-35Nb-7Zr-5Ta was used, i.e., approximately 5.34 MPa. When using CoCr, the lowest contact stress occurred, i.e., approximately 4.2 MPa, for a difference of approximately 21%. The same trend occurred for von Mises stress and deflection on the cement mantle. Based on these simulation results, the CoCr material is the best for reducing stress and displacement in the cement mantle.

## Figures and Tables

**Table 1:** Mechanical properties of the material used.

Materials	Modulus of Elasticity (MPa)	Poisson’s Ratio
Cortical bone [16,17,18]	17,000	0.3
PMMA [17,18,19]	2000–2300	0.3
UHMWPE [18,19,20]	690–945	0.45
SS316L [21]	193,000	0.3
CoCr [22]	230,000	0.3
Ti-35Nb-7Zr-5Ta [23]	55,000	0.33
Ti-6AI-4V [22]	110,000	0.32
